# A semi-automated genome annotation comparison and integration scheme

**DOI:** 10.1186/1471-2105-14-172

**Published:** 2013-06-01

**Authors:** Zhe Liu, Hongwu Ma, Igor Goryanin

**Affiliations:** 1 Computational Systems Biology and Bioinformatics, School of Informatics, University of Edinburgh, Informatics Forum, 10 Crichton Street, Edinburgh, EH8 9AB, UK; 2 Key Laboratory of Systems Microbial Biotechnology, Tianjin Institute of Industrial Biotechnology, Chinese Academy of Sciences, Tianjin, China; 3 Biological Systems Unit, Okinawa Institute of Science and Technology, 1919-1 Tancha, Onna-son, Kunigami-gun, Okinawa, Japan

**Keywords:** Genome annotation comparison, Genome annotation determination, Automated annotation services

## Abstract

**Background:**

Different genome annotation services have been developed in recent years and widely used. However, the functional annotation results from different services are often not the same and a scheme to obtain consensus functional annotations by integrating different results is in demand.

**Results:**

This article presents a semi-automated scheme that is capable of comparing functional annotations from different sources and consequently obtaining a consensus genome functional annotation result. In this study, we used four automated annotation services to annotate a newly sequenced genome--*Arcobacter butzleri* ED-1. Our scheme is divided into annotation comparison and annotation determination sections. In the functional annotation comparison section, we employed gene synonym lists to tackle term difference problems. Multiple techniques from information retrieval were used to preprocess the functional annotations. Based on the functional annotation comparison results, we designed a decision tree to obtain a consensus functional annotation result. Experimental results show that our approach can greatly reduce the workload of manual comparison by automatically comparing 87% of the functional annotations. In addition, it automatically determined 87% of the functional annotations, leaving only 13% of the genes for manual curation. We applied this approach across six phylogenetically different genomes in order to assess the performance consistency. The results showed that our scheme is able to automatically perform, on average, 73% and 86% of the annotation comparison and determination tasks, respectively.

**Conclusions:**

We propose a semi-automatic and effective scheme to compare and determine genome functional annotations. It greatly reduces the manual work required in genome functional annotation. As this scheme does not require any specific biological knowledge, it is readily applicable for genome annotation comparison and genome re-annotation projects.

## Background

Currently, researchers can use parallel sequencing technologies to obtain whole genome sequences with relatively low cost and in a short time [1,2]. Determination of the gene functions in a genome becomes a bottleneck for further functional analysis. To resolve this issue, several projects have been designed to provide automated genome annotation services [1,3-6], such as IMG/ER (Integrated Microbial Genome Expert Review system) from the Joint Genome Institute [4], the National Microbial Pathogen Data Resource’s RAST (Rapid Annotation using Subsystems Technology) server [5], JCVI (J. Craig Venter Institute) annotation service [7] and University of Maryland’s IGS (Institute for Genome Sciences) annotation engine [8]. These services can greatly reduce the cost and human efforts needed for annotating genome sequences [3,9,10]. However, they often generate different results from different annotation methods and it is difficult to compare them and decide which one is more suitable [10,11]. Genome annotation may refer to structural annotation and functional annotation. In this paper we focused on functional annotation only. There are two types of functional annotation differences: term difference (using different terms to describe the same biological function) and inconsistent annotation (different biological function). The existence of a large number of term differences makes it very difficult to automatically compare different genome annotation results by programming. Researchers attempted to solve this issue by introducing controlled vocabularies, such as Gene Ontology (GO) [12]. However, only one out of the four annotation services includes GO terms, making it difficult to standardise terms by GO IDs. In addition, EC and gene symbol are other standardisation approaches to deal with term differences. In this study, we constructed a baseline method using database ID comparison (EC and gene symbol) and annotation text matching. We found that it can compare only 45% of the annotations for *Arcobacter butzleri* ED-1 (Arc-ED). Therefore, it is desirable to design a system capable of automatically standardising term differences at a high percentage rate. For inconsistent annotations, we need to decide which annotation is more likely to be correct [6,7,9]. The best approach is through experimental evidence; however it is difficult to gain experimental evidence for all inconsistent annotations in a newly sequenced genome. In this paper, we adopted a compromising approach by using majority supported annotations as the consensus annotations.

Some attempts have been made to solve the issue of annotation difference. Takeya *et al.* proposed a prototypical scheme to compare different annotations and decide on consensus annotations for a mouse genome annotation project [13]. This scheme aims to eliminate the uninformative annotations (annotations without indication of clear biological functions) and compare annotations with the sequence similarity search results through several steps. However, a significant amount of human effort is required to reconcile term differences and choose the accurate annotations. As a departure from prior research, we propose a semi-automated approach that first compares annotations and then obtains consensus annotations based on the comparison results. First, we employed information retrieval techniques and introduced gene synonym lists to standardise the term differences. Then we compared the gene annotations in pairs. Lastly, based on the annotation comparison results, we designed an annotation decision approach, which employs majority voting to obtain a consensus annotation. The experimental results show that our scheme is able to compare and determine 87% and 87% of the annotations, respectively. Moreover, when we applied our scheme on six phylogenetically different genomes, it achieved 73% of the annotation comparison rate and 86% of the annotation determination rate.

## Methods

### Annotation comparison

In this study, we used the genome of *Arcobacter butzleri* ED-1 (Arc-ED), a newly sequenced *Epsilonproteobacterium*, to demonstrate our method [14]. The Arc-ED genome sequence was obtained from GenBank with accession number AP012047. Four automated annotation services were employed to obtain the annotations. Then we merged the structural annotation results as follows:

1) When the structural annotation results have the same stop site, we consider them to be the same genes;

2) When the length of a gene is less than 150bp, we remove it.

In this way, we obtained 2178 coding genes totally. The genome annotations from the four automated annotation services are used in the following sections for genome annotation comparison and determination tasks.

It was reported that there are a great amount of term differences in automated annotation results [10]. We conducted a baseline comparison to demonstrate the prevalence of differing annotations. The four automated genome annotation results are compared in pairs, merely by database IDs and identical text matching of the gene function. We extracted database IDs and lower-cased the annotations. Then we compared the annotations, using the following rules:

1) If the database IDs (ECs, gene symbols, Pfam IDs, TIGRfam IDs, COG IDs) are the same, then we considered them to be the same annotations. Here, identical database IDs denote that at least one type of the database IDs is exactly the same, and this rule applies to the following comparison as well;

2) If annotation texts or terms (Pfam terms, TIGRfam terms, COG terms) are identical, then we considered them to be the same annotations;

3) If one annotation is an uninformative annotation (such as “hypothetical protein” and “conserved hypothetical protein”) and the other annotation has functional annotation, then we considered them as different annotations;

4) The matching relationship can be transferred. For example, if IMG has the same annotation as both IGS and JCVI, then annotations from IGS and JCVI are considered to be the same.

It is shown that the baseline procedure can deal with only 45% of the annotations automatically. To increase the automated comparison rate, we discovered that there are three types of term differences, they are:

1) Text variants (variants coming from different word forms or stop words);

2) Synonyms and abbreviations;

3) Functional annotation variants (variants originating from orthologous annotations in different organisms).

**Figure 1:**
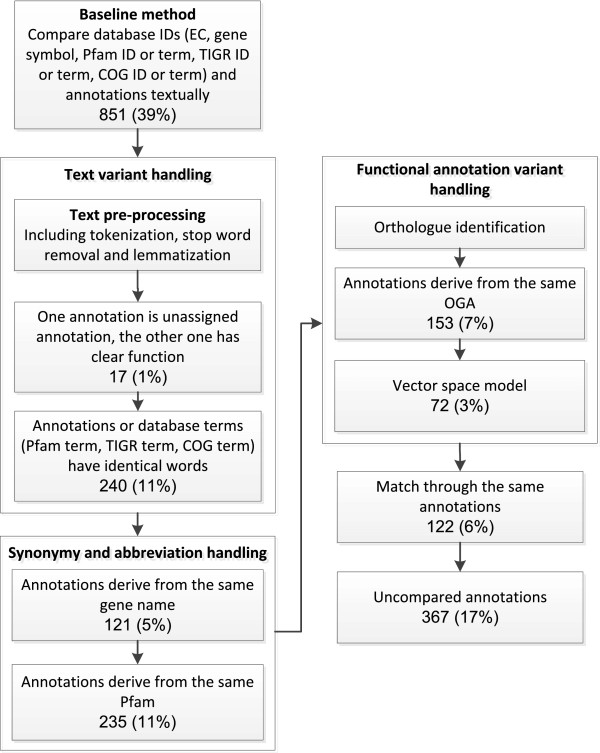
**Flowchart of the gene annotation comparison procedure.** The boxes represent the processes used to compare the annotations and the figures in each box denote the annotation comparison number and rate.

In the following paragraphs, we introduce the approaches to handle term differences and the annotation comparison scheme in detail. The datasets and Python code used to carry out the following procedures can be seen in the supplementary files (Additional file 1: code and datasets.zip). Here we take the comparison result between IGS and RAST to illustrate the entire comparison scheme, as shown in Figure 1. The same procedure applies to the pair-wise comparison between any two of the four annotations as well. In this case, we performed the comparisons six times. We ran the baseline comparison and the result showed that it is able to deal with 39% of the annotations between IGS and RAST.

### Text variant handling

In this section, we applied techniques developed in the domain of information retrieval to deal with text variants. The first example, from Table 1, shows two annotations, using “kinases”/“kinase”, “proteins”/“protein”, respectively. They indicate the same biological function but use different texts. For this condition, we used text pre-processing techniques from information retrieval to convert gene annotations into citation word lists. Gene annotations are processed by tokenization, stop word removal and lemmatization. The Python package ‘NLTK’ is used to perform these jobs [15].

#### Tokenization

Tokenization aims to chop annotations into words and discard certain characters, such as punctuation. For example, tokenization turns the annotation “DNA polymerase III, beta subunit” into a list with elements “DNA”, “polymerase”, “III”, “beta” and “subunit”. It is observed that changing word order has little effect on biological function expression, therefore unordered annotation word lists are used in the following analyses.

#### *Stop word removal*

Stop words are words with little meaning and discriminative power in documents. In information retrieval

**Table 1:** Examples of three types of term difference

**Term difference**	**Annotation 1**	**Annotation 2**
Text variant	cAMP-binding proteins - catabolite gene activator and regulatory subunit of cAMP-dependent protein kinases	putative cAMP-binding protein - catabolite protein activator and regulatory subunit of cAMP-dependent protein kinase
Synonym and abbreviation	RIP metalloprotease	Membrane-associated zinc metalloprotease
Functional expression variant	phosphoribosyl-ATP pyrophosphatase/phosphoribosyl-AMP cyclohydrolase	phosphoribosyl-ATP diphosphatase

 research, there is a frequently used stop word list which contains common words, such as “the” and “an”. We found that the common words in gene annotations differ from these stop words. For example, “putative” in “putative membrane protein” indicates little biological function, which ought to be removed for comparison. We constructed a biological stop word list for this study, which is available in the supplementary files (Additional file 2: stop word list.pdf).

#### Lemmatization

It is known that words have inflected forms in order to carry out different roles in annotations. For instance, “proteins” and “protein” derive from the same lemma but are textually different. In this study, we used WordNet to obtain the lemmas of the words [16].

#### Annotation comparison

In the end, we used the following criteria to compare the annotations in pairs:

1. When only one annotation is an uninformative annotation (annotations without indication of clear biological functions, such as “clustering with…” and “conserved domain protein”), the relationship between the two annotations is considered as “one hypothetical protein and one clear function”,

2. We transformed the annotations into word lists and when both of the annotations have the same word list, we considered them to be the same annotation.

The results from Figure 1 show that the above procedure can deal with 1% and 11% of the annotations for the comparison between IGS and RAST.

### Synonym and abbreviation handling

Synonyms and abbreviations are related to the differences deriving from the usage of different descriptions of the same database entry. Genes with same biological functions may have different gene names, for example, “RIP metalloprotease”, “Membrane-associated zinc metalloprotease” and “rseP” come from the same database ID and they denote the same function. In addition, the Pfam database has Pfam term, Pfam ID and Pfam abbreviation; for instance, “PALP”, “pfam00291” and “Pyridoxal-phosphate dependent enzyme” come from the same Pfam database entry and indicate the same Pfam domain information. However, these annotations are textually different, which stop computers from treating them as the same annotation. In this case, we employed the gene symbol data [17] from the NCBI database and Pfam data from the Pfam database [18] as synonym lists to assist annotation comparison. It is known that a protein may have multiple domains to carry out biological functions. We only consider annotations with entirely identical domain information as the same annotations, *e.g.* IMG annotated gene 15 with two Pfam domains pfam00126 and pfam03466, therefore, if RAST has both of the domains then they are considered to be the same annotation. This procedure can improve the automated comparison rate between IGS and RAST by 16%, which brings it to 67%.

#### Functional annotation variant handling

Since different annotation services use variant reference genome databases and gene annotation algorithms to assign protein functions, we may be presented with variant annotations, which are the best hits, coming from different organisms. It is believed that orthologous gene annotations (OGA) are derived from the same ancestor and have similar functions. However, when we check the annotation results, it is difficult to figure out where the annotations come from and assert whether they refer to the same biological functions or not. For example, “phosphoribosyl-ATP pyrophosphatase/phosphoribosyl-AMP cyclohydrolase” and “phosphoribosyl-ATP diphosphatase” come from the same OGAs and are assumed to carry out similar functions. A holistic collection of OGAs can help us to link annotations from different organisms together. In the following study, we used OMA (the Orthologous MAtrix project), a comprehensive collection of OGAs, as a bridge to compare different annotations [19]. OMA first launches all-against-all alignments between two genomes using the Smith-Waterman algorithm, then the “symmetrical best hits” are considered as orthologous genes [20]. The data from OMA are pair-wise Bidirectional Best Hits (BBHs). In our study, we transformed them into an OGA collection by grouping all of the annotations mapped to the investigated gene. We downloaded OMA data and extracted the bacteria and archaea gene annotations for the following studies.

#### Orthologue identification

In order to use OGAs from OMA to compare the annotations, we should figure out the corresponding OGAs for a specific Arc-ED gene. In our study, we used *A. butzleri* strain RM4018 (Abu), the phylogenetically closest organism to Arc-ED, to map the OGAs to Arc-ED genes. The linkage between Arc-ED and Abu gene annotations is built up using Bidirectional Best Hit (BBH), a standard approach to identify two genes with very strong similarities as orthologues. We conducted a BLAST search between Arc-ED and Abu to find BBHs by requiring the BLAST E-value to be less than 1E-5. The Abu genome is included in the OGA data and we mapped 1484 Arc-ED genes to OGAs, consequently.

#### OGA-based annotation comparison

We used two approaches to compare gene annotations with their corresponding OGAs. Primarily, gene annotations are considered to be the same when both of them have the same words as any gene annotation in their corresponding OGAs. In this way, we compared a further 7% of the annotations between IGS and RAST as shown in Figure 1.

**Figure 2:**
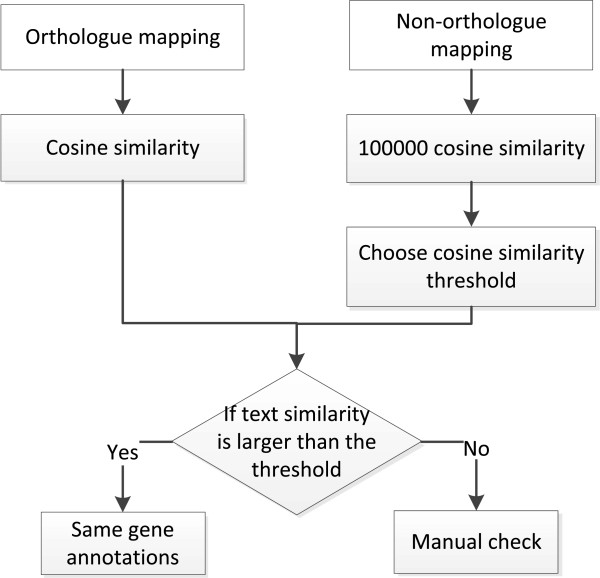
Flowchart of similarity cut-off determination for vector space model.

Secondly, for gene annotations unable to be compared by the above procedure, we used a vector space model to evaluate the similarities between gene annotations and OGAs. Then, we set a threshold and used it to assign the same gene annotations. When both of the two automated gene annotations have sufficiently high similarity with OGAs, they are regarded as the same gene annotations. The complete process of this method is shown in Figure 2. We described the vector space model first, and then the procedure to establish the similarity cut-off. In this study, Arc-ED gene annotations and OGAs are viewed as vectors, with each element representing word frequency. The vector space model is used to capture the text similarity between gene annotations and OGAs. In the meantime, we used term frequency – inverse document frequency (*tf-idf*), a classic word weighting scheme, to assign various weights to different words [21]. The intuition is that the importance of a word, *w*, can be approximated by the product of word frequency, *tf*_*w*_, and the inverse document frequency, *idf*_*t*_. Inverse document frequency is denoted by the logarithm value of dividing the total number of documents (annotations) by the number of documents (annotations) containing the word. It aims to scale the weights of the common words, such as “protein” and “function” in the annotations. The word weight for an Arc-ED gene annotation *G*_*w*_ is defined in the following equation [21]:

$$G_{w}=tf_{w,G}.\log\frac{N_{G}}{df_{w,G}}$$

where *G*_*w*_ is the gene annotation word weight vector, *tf*_*w,G*_ is the gene annotation word frequency, *N*_*G*_ is the number of gene annotations in the entire collection

 and *df*_*w,G*_ is the number of gene annotations containing word *w*.

As for OGA, a modified equation shown below is used to consider document length and repeated words (It should be noted that OGA in the following equation refers to the corresponding OGA for the specific Arc-ED gene.):

$$O_{w}=\frac{tf_{w,O}}{tf_{w,O}+\frac{1.5L_{O}}{\mathrm{avg}.\mathrm{len}}}.\log\frac{N_{O}}{df_{w,O}}$$

where *O*_*w*_ is the OGA word weight vector, *tf*_*w,O*_ is the OGA word frequency, *L*_*O*_ is the OGA word length, *avg.len* is the OGA average word length, *N*_*O*_ is the number of OGAs and *df*_*w,O*_ is the number of OGAs containing word *w*.

Moreover, cosine similarity is used to capture the similarity between an annotation and an OGA, as below [21]:

$$s\left(G,O\right)=\frac{\sum_{w}G_{w}O_{w}}{\sqrt{\sum_{w}G_{w}^{2}.\sum_{w}O_{w}^{2}}}$$

where *s*(*G,O*) is the weighted cosine similarity, *G*_*w*_ is the gene annotation word weight vector and *O*_*w*_ is the OGA word weight vector.

For instance, we have two annotations for gene 978 from IGS and IMG results. They are ‘glutamyl-tRNA(Gln) amidotransferase subunit A (Glu-ADTsubunit A)’ and ‘aspartyl/glutamyl-tRNA(Asn/Gln) amidotransferase subunit A (EC 6.3.5.-)’, respectively. They are different in terms of text. When we compared them with OGA, we discovered that OGA contains a mixture of texts, such as ‘glutamyl-tRNA(Gln)’, ‘amidotransferase’ and ‘Aspartyl/glutamyl-tRNA(Asn/Gln)’. We know that this gene should carry out a function related to amidotransferase, therefore, the more ‘amidotransferase’ appears in IGS and IMG annotations, the more weight this word should have. In the meantime, we find out that OGA contains repeated words, such as ‘glutamyl-tRNA’, ‘Gln’, ‘amidotransferase’ and ‘subunit’. The first three words are specific to this OGA and less likely to appear in the OGAs of the other genes, thus they should have more weight. ‘subunit’ is a common word which appears across annotations with different functions and should have less weight. The similarity between IGS and OGA is computed as 0.901 through this weighting scheme.

Furthermore, we need to know the cut-off in order to assign different annotations to the same function. The entire procedure to determine the cut-off is shown in Figure 2. First, we created the non-orthologous dataset by randomly reshuffling the mapping between OGAs and gene annotations. In the meantime, we controlled that the OGA for a gene annotation won’t be the same as the OGAs of its orthologue. For example, supposing that Arc-ED gene *i* is mapped to OGA through Abu gene *j* in the orthologous dataset, all of the mappings from Arc-ED gene *i* to OGAs through the annotations different from the Abu gene *j* annotation are allowed in the non-orthologous dataset. Subsequently, we computed 100000 non-orthologue similarity scores. We used the top 100th similarity score, which is around 0.7, as the similarity score cut-off. This indicates that when the similarity score is larger than 0.7, the probability of a non-orthologous annotation assumed to be the orthologous annotations is below 0.001. Hence, gene annotations with similarity score both larger than 0.7 with an OGA are considered to derive from the same OGA and have the same function. The results between IGS and RAST from Figure 1 show that the OGA-based similarity comparison method can deal with a further 3% of the annotations.

### Matching between comparison results

Finally, same gene annotations are used to map the annotations unable to be compared in the previous sections. For example, when the IGS gene annotation is the same as the IMG and JCVI annotations, they are considered to be the same annotations as well. This process can compare 6% of the IGS and RAST annotations and altogether we can compare 1811 out of 2178 gene annotations automatically, accounting for 83% of the coding sequences. 367 (17%) gene annotations can not be compared automatically and thus they are left for manual examination. It is shown that our method is significantly better than the baseline method, which increases the annotation comparison rate from 39% to 83%.

### Paired annotation comparison results

We show the step-by-step paired comparison results between any two of the four automated annotations in Table 2. The numbers represent the percentage of the annotations automatically compared in each step. It is shown that all of the automated comparison rates between any two of the annotations exceed 80%. On average, text variant handling (one HP another non-HP, annotation and database term) can compare around 22% of the annotations; synonyms and abbreviation handling (gene symbol entry, Pfam entry) is able to deal with around 11% of the annotations; functional annotation variant handling procedure (orthologue, vector space model) can compare around 6% of the annotations. Lastly, matching procedure is able to make a further 2% of the annotation comparison.

### Overall annotation comparison results

The overall comparison results show that our procedure can handle 87% of the annotations. Only 13% of the annotations could not be compared automatically and needed manual comparison.

**Table 2:** Paired comparison results between automated annotation services

	**IGS vs IMG**	**IGS vs JCVI**	**IGS vs RAST**	**IMG vs JCVI**	**IMG vs RAST**	**JCVI vs RAST**
Baseline	62%	51%	39%	46%	40%	32%
One HP another non-HP	1%	1%	1%	2%	2%	1%
Annotation and database term	23%	20%	11%	27%	14%	30%
Gene symbol entry	1%	2%	5%	1%	4%	5%
Pfam entry	3%	7%	11%	6%	10%	8%
Orthologue	1%	2%	7%	3%	7%	6%
Vector space model	1%	2%	3%	1%	2%	2%
Matching	1%	3%	6%	1%	4%	1%
Overall result	93%	88%	83%	87%	83%	85%

Baseline: baseline annotation comparison result; One HP another non-HP: one annotation is a ‘hypothetical protein’ and another one has a characterised function; Annotation and database term: two annotations have the same annotation text or database terms; Gene symbol entry: annotations are derived from the same gene symbol entry; Pfam entry: annotations are derived from the same Pfam entry; Orthologue: annotations are derived from the same OGA; Vector space model: vector space model comparison; Matching: matching between comparison results; Overall result: total number of gene annotations compared.

### Annotation determination

**Figure 3:**
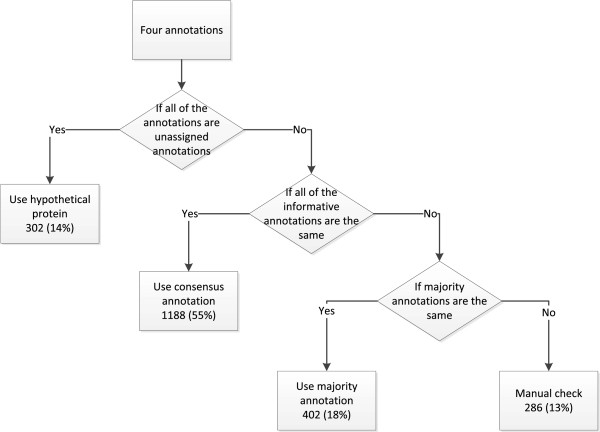
Flowchart of gene annotation determination procedure.

In this section, we used the annotation comparison result and constructed a decision tree (Figure 3) to derive a consensus genome annotation result. Without any biological evidence, we could not assert which annotation was more reliable than the others. Therefore we assumed that the gene annotations with the majority supports ought to be of high 

1) We first check if all of the annotation results are uninformative annotations, such as “uncharacterized annotation” and “hypothetical protein”. In this case, we choose “hypothetical protein” as the consensus annotation.

2) We then check if all of the annotation results are the same or derive from the same OGA.

3) In the last step, we check whether the annotations that agree with each other form a majority, and then we use them as the consensus annotation.

4) The rest of the annotations unable to be determined by the above processes need to be manually reviewed for annotation determination. reliability. The decision rules are shown as follows:

**Table 3:** Discrepant annotations between automated annotation services and consensus annotation

**Annotation service**	**Number of discrepant annotations**
IGS	74
IMG	122
JCVI	133
RAST	134

The annotation determination result is promising in that it is able to determine a large amount of the annotations automatically. Altogether, we determined the annotations for 1892 (87%) genes, leaving only 286 (13%) genes for manual review. Of the entire genome, 302 (14%) genes use “hypothetical protein” annotations as the consensus annotations. In the meantime, 1188 (55%) annotations are the same among four automated annotations and 402 (18%) annotations are supported by majority of the results.

## Result and discussion

In this section, we carried out three experiments to evaluate our approach. First, we attempted to study if our approach is able to improve the *Arcobacter butzleri* ED-1 genome annotation by integrating different annotation results. Second, we are interested in discovering whether the performance of our approach is consistent across phylogenetically different genomes. Third, we tried to study if the consensus annotation obtained is consistent with the literature-based annotation from EcoCyc.

### Discrepant annotations between the consensus annotation and four annotation services

We manually compared the four automated annotation results of *Arcobacter butzleri* ED-1 with the consensus annotation to evaluate the quality of the annotation obtained. There are around 100 inconsistent annotations for different annotation results, as shown in Table 3. IGS has the least and RAST has the most discrepant annotations with the consensus annotation: 74 and 134 genes respectively. We presented an example to showcase the discrepant annotations between the automated annotations and consensus annotation in Table 4. For instance, IGS, IMG, JCVI and RAST are the only services to provide characterised functional annotations for genes 76, 2119, 547 and 2176, respectively. In this case, merging the different annotation results can effectively increase the coverage of consensus annotation.

Since there is little biological evidence to evaluate the annotation quality, a reasonable approach to verify an annotation is by bioinformatic evidence. We used BLAST [22], Pfam [22], TMHMM [23] and TIGRfam [24] search results as the bioinformatic evidence. The intuition is that when there are one or more evidence supporting a certain biological function, we consider it to be the reliable annotation. We manually compared the RAST annotation with the consensus annotation. The cut-off settings for different algorithms are as follows: BLASTP (e-value < 1E-5), Pfam (e-value < 0.001), TIGRfam (e-value < 0.001). Some results are shown in Table 5 and the complete data can be found in the supplementary files (Additional file 3: discrepant annotations.xls).

We classified the different annotations into four categories. The first category is that the consensus annotation is proper; the second category denotes that none of the results are appropriate due to conflicting evidence; the third category is that the RAST annotation result is more reliable; the fourth category is that we could not decide which annotation is more reliable due to insufficient evidence.

Among the 134 genes with different annotations, 25 genes have more precise or reliable annotations in the consensus annotation (gene 44 and 1333 in Table 5). For example, gene 44 is annotated “hypothetical protein” by RAST and “membrane transport family protein” by the consensus result, respectively. When we carried out Pfam, TIGRfam, BLAST and TMHMM searches, it was shown that this protein contains trans-membrane helices. Therefore, it should be designated as “membrane transport family protein”. Moreover, we discovered two genes which neither of the annotations is accurate (gene

**Table 4:** Examples of discrepant annotations between automated annotation services and consensus annotation

**Gene ID**	**IGS**	**IMG**	**JCVI**	**RAST**	**Consensus annotation**
76	putative membrane protein	hypothetical protein	conserved hypothetical protein	hypothetical protein	Putative membrane protein
2119	hypothetical protein	ABC-type Co2+ transport system			ABC-type Co2+ transport system, periplasmic component
547	hypothetical protein	hypothetical protein	prepilin-type N-terminal cleavage/methylation domain protein	hypothetical protein	prepilin-type N-terminal cleavage/methylation domain protein
2176	hypothetical protein	hypothetical protein	conserved hypothetical protein	FAD/FMN-containing dehydrogenases	FAD/FMN-containing dehydrogenases

**Table 5:** Discrepant annotations between RAST and consensus annotation

**Gene ID**	**RAST annotation**	**Consensus annotation**	**Reliable annotation**	**Bioinformatic evidence**
44	hypothetical protein	membrane transport family protein	consensus	Pfam, TIGRfam, BLAST, TMHMM
1333	protein of unknown function DUF481	Putative salt-induced outer membrane protein	consensus	BLAST
77	hypothetical protein	tat (twin-arginine translocation) pathway signal sequence domain protein	neither	NADH dehydrogenase, FAD-containing subunit (BLAST)
2081	conserved hypothetical protein	Methyltransferase domain.	neither	Tellurite resistance protein TehB (pFam)
982	histidine kinase	bacterial extracellular solute-binding proteins, family 3 family protein	RAST	two-component sensor histidine kinase (BLAST)
1900	hypothetical protein	N- methylation	RAST	hypothetical protein (BLAST)
183	conserved hypothetical protein	putative membrane protein	not enough evidence	
212	conserved hypothetical protein	transcriptional regulator, Spx/MgsR family	not enough evidence	

 77 and 2081 in Table 5). For instance, RAST annotated gene 77 as “hypothetical protein” and the consensus annotation used “tat (twin-arginine translocation) pathway signal sequence domain protein” as the annotation. Neither of the annotations is reliable because BLASTP and Pfam results show that the function is “NADH dehydrogenase, FAD-containing subunit”. 9 gene annotations in RAST have more bioinformatic evidence than the consensus result (gene 982 and 1900 in Table 5). In addition, we observed that two genes are annotated as “hypothetical protein” in RAST but are assigned characterized functions in the consensus annotation (gene 183 and 212 in Table 5). We found that and none of the bioinformatic tools was able to figure out the characterised functions. Lastly, we had insufficient bioinformatic evidence to determine the annotations for 98 genes. The results demonstrated that the consensus annotation tends to have more bioinformatic evidence than RAST annotation. Therefore, our approach is able to improve the automated annotation for the *Arcobacter butzleri* ED-1 genome by integrating different annotations.

**Figure 4:**
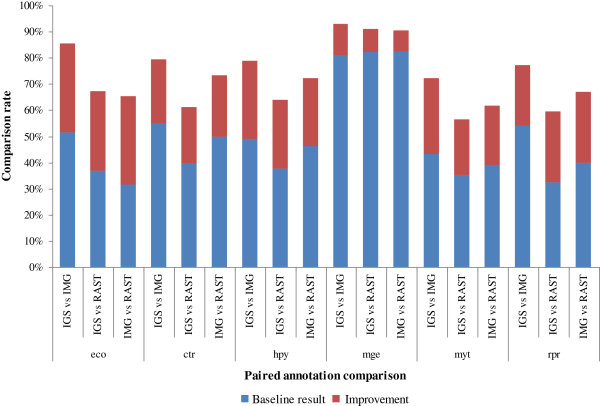
**Automated annotation comparison results for six genomes.** eco, ctr, hpy, mge, mtu, rpr stand for the automated genome annotation comparison results for *Escherichia coli* K-12 MG1655, *Chlamydia trachomatis* D/UW-3/CX, *Helicobacter pylori* 26695, *Mycoplasma genitalium* G37, *Mycobacterium tuberculosis* H37Rv and *Rickettsia prowazekii* Madrid E, respectively. These abbreviation representations apply to the following sections as well.

**Table 6:** Genome annotation comparison results for six genomes

**Organism**	**Paired comparison**	**Baseline**	**Our method**	**Improvement**
eco	IGS vs IMG	52%	86%	34%
	IGS vs RAST	37%	68%	31%
	IMG vs RAST	32%	66%	34%
ctr	IGS vs IMG	55%	79%	24%
	IGS vs RAST	40%	61%	21%
	IMG vs RAST	50%	73%	23%
hpy	IGS vs IMG	49%	79%	30%
	IGS vs RAST	38%	64%	26%
	IMG vs RAST	46%	72%	26%
mge	IGS vs IMG	81%	93%	12%
	IGS vs RAST	82%	91%	9%
	IMG vs RAST	82%	90%	8%
myt	IGS vs IMG	43%	72%	29%
	IGS vs RAST	35%	56%	21%
	IMG vs RAST	39%	62%	23%
rpr	IGS vs IMG	54%	77%	23%
	IGS vs RAST	32%	59%	27%
	IMG vs RAST	40%	67%	27%

### Performance validation on six phylogenetically different genomes

To evaluate the performance of our method, we applied our scheme to six prokaryote genomes: *Escherichia coli* K-12 MG1655, *Chlamydia trachomatis* D/UW-3/CX, *Helicobacter pylori* 26695, *Mycoplasma genitalium* G37, *Mycobacterium tuberculosis* H37Rv and *Rickettsia prowazekii* Madrid E. We noticed that the JCVI annotation service was temporarily unavailable, so we only used the rest of the annotation services to annotate the genomes. The paired comparison results for the six different genomes in Figure 4 and Table 6 show that the baseline method can compare, on average, 49% of the annotations. Our scheme is able to further improve the comparison rate to around 73%. The best result is obtained for the *Mycoplasma genitalium* G37 genome. 92% of the gene annotations can be compared.

**Figure 5:**
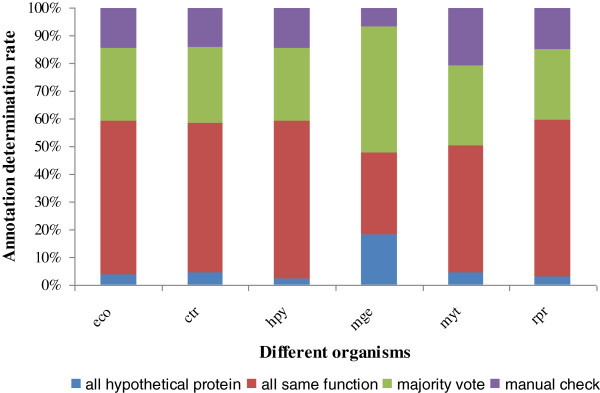
Automated annotation determination results for six genomes.

We then followed the same rules described in section 2 for the annotation determination task. The six genome determination results are shown in Figure 5. Overall, the annotation determination scheme can automatically determine 86% of the annotations, leaving 14% of the annotations for manual check. Meanwhile, around 50% of the consensus annotations come from the annotations supported by all of the annotations. Around 30% of the consensus annotations are derived from majority-supported annotations. These results suggest that our approach is able to achieve high percentage of annotation comparison and determination rates.

### Annotation quality evaluation on the *E.coli* genome

To evaluate the quality of the consensus annotation, we compared the *E.coli* consensus annotation with the literature-based annotation result obtained from the EcoCyc database [25]. EcoCyc identified 4499 genes for the genome of *E.coli* and the consensus result recognized 4394 genes. 4019 genes are shared between the two results and our scheme determined 3443 (86%) gene annotations. We then carried out the manual comparison work between the consensus annotation and EcoCyc annotation. The complete data are attached in

 the supplementary files (Additional file 4: comparison between consensus annotation and EcoCyc annotation.xls). Our scheme is able to identify 3104 (77%) genes with the same functional annotations as EcoCyc. In the meantime, only 339 (9%) genes in consensus result have different annotations from EcoCyc result. It is shown that the consensus annotation obtained is able to identify high percentage of the annotations as same as EcoCyc result.

To examine if our integration scheme really produced better results than the individual services, we also compared the annotation results between IMG and EcoCyc. We chose IMG because it carried out comprehensive bioinformatic search in the annotation process [4]. The result shows that there are 352 different annotations between them, slightly bigger than the difference between EcoCyc and the consensus annotation. This result indicates that the integration scheme has improved the annotation quality.

## Conclusions

In this paper, we present a semi-automated procedure to compare and determine genome annotations from different annotation sources. The contributions of this approach are divided into two parts:

1) We designed an automated annotation comparison and determination approach which achieves high (greater than 80%) annotation comparison and determination rates for the genome of *Arcobacter butzleri* ED-1. The performance is consistent across six phylogenetically different genomes (on average 73% and 86% with respect to annotation comparison and determination rates). The evaluation results show that our approach is able to improve the annotation quality and only 9% of the annotations are different from EcoCyc result with respect to the *E. coli* genome.

2) We constructed a biological stop word list which can be used in genome annotation comparison research.

There is no need of specific biological knowledge in the annotation comparison and determination processes, therefore our approach can be readily applied to other genome re-annotation/comparison projects.

## Abbreviations

GO: Gene ontology; OGA: Orthologous gene annotations; IGS: Institute for genome sciences annotation service; IMG/ER: Integrated microbial genome expert review system; JCVI: J. Craig Venter Institute annotation service; RAST: Rapid annotation using subsystems technology; Arc-ED: *Arcobacter butzleri* ED-1; Abu: *Arcobacter butzleri* strain RM4018; COG: Clusters of Orthologous Groups; TIGR: The Institute for Genomic Research

## Competing interests

The authors declare that they have no competing interests.

## Authors’ contributions

ZL designed and performed the experiments and wrote the manuscript. HM provided the overall project guidance, critical review and gave advice on the experiment and validation. IG gave advice on the experiment and helped to draft the manuscript. All authors read and approved the manuscript.

## Supplementary Material

Additional file 1 **The code and datasets: file contains the python code and related datasets used in our research.** We used two datasets in our research. (1). ‘pfam_mapped_dic.csv’ file contains the Pfam IDs obtained from Pfam database; (2) ‘gene_info_dic.csv’ file contains the gene symbol data obtained from Entrez gene database; (3) ‘database data’ folder contains the automated annotation results and the OMA data to run the program. Click here for file

Additional file 2 **Stop word list: file contains a genome annotation relevant stop word list constructed in this research.** This stop word list can be easily applied to other genome annotation comparison research. Click here for file

Additional file 3 **Discrepant annotations: file contains the discrepant annotations between four annotations and the consensus annotation.** The bioinformatic evidence is presented as well. Click here for file

Additional file 4 **Comparison between consensus annotation and EcoCyc annotation: file contains the annotation comparison results between the *E.coli *consensus annotation and EcoCyc annotation.** Click here for file
