# Supplementary material for: A semi-automated genome annotation comparison and integration scheme
Source: BMC Bioinformatics. 2013 Jun 1;14:172. doi: 10.1186/1471-2105-14-172 (PMC3680241; doi:10.1186/1471-2105-14-172)
Supplement: Additional file 2 — Stop word list: file contains a genome annotation relevant stop word list constructed in this research. This stop word list can be easily applied to other genome annotation comparison research. [file 1471-2105-14-172-S2.doc]

# Additional File 2

## Article title: A semi-automated genome annotation comparison and integration scheme

## Authors: Zhe Liu, Hongwu Ma and Igor Goryanin

# 1. Genome annotation relevant stop word list

When we compared the annotations from different sources, we constructed a genome annotation relevant stop word list as shown below. This stop word list can be easily applied to other genome annotation comparison/re-annotation tasks.

n-terminal

subfamily

relate

-

cluster

uncharacterized

biosynthetic

biosynthesis

molecular

pathway

system

c-terminal

activity

binding

containing

possible

have

probable

for

region

unknown

to

bind

bacterial

ec

in

unkonwn

superfamily

subunit

component

and

enzyme

associate

type

involve

function

consistdomain-containing

putative

probable

predict

family

translate

translation

transcription

signal

protein

[

]

(

)

--

;

:

,

/

or

with

the

contain

of

product

related

domain

an

|

.

conserve

#

response

chain
