# Supplementary material for: A semi-automated genome annotation comparison and integration scheme
Source: BMC Bioinformatics. 2013 Jun 1;14:172. doi: 10.1186/1471-2105-14-172 (PMC3680241; doi:10.1186/1471-2105-14-172)
Supplement: Additional file 1 — The code and datasets: file contains the python code and related datasets used in our research. We used two datasets in our research. (1). ‘pfam_mapped_dic.csv’ file contains the Pfam IDs obtained from Pfam database; (2) ‘gene_info_dic.csv’ file contains the gene symbol data obtained from Entrez gene database; (3) ‘database data’ folder contains the automated annotation results and the OMA data to run the program. [file 1471-2105-14-172-S1.zip › Additional file 1 code and datasets/readme.doc]

This README file discusses the Python code and datasets associated with the paper:

## Article title: A semi-automated genome annotation comparison and integration scheme

## Authors: Zhe Liu, Hongwu Ma and Igor Goryanin

This directory contains supporting python codes and datasets to perform annotation comparison and determination tasks.

## Datasets

The ‘datasets’ folder contains the data used in our research, in particular, the contents of data used are listed below:

1. This file ‘pfam_mapped_dic.txt’ contains the Pfam dictionary constructed from Pfam database ;
2. This file ‘gene_info_dic.txt’ contains the gene symbol dictionary constructed from Entrez gene database .
3. The ‘database data’ folder contains the automated annotation results and the OMA data for each specific genome.

## Python Code

The ‘code’ folder contains supporting Python code to pre-process, compare and determine genome annotations from multiple annotation sources. It should be noted that BLAST software should be installed prior to the usage of this code. BLAST can be found on NCBI BLAST website (<ftp://ftp.ncbi.nlm.nih.gov/blast/executables/blast+/LATEST/>). ‘pre-treat.py’ and ‘pre.py’ are used to format and retrieve the OGA annotations from OMA database. ‘annoComp.py’, ‘annoDeter.py’ and ‘pre.py’ are used to compare the annotations and derive a consensus annotation result based on the comparison result. The detailed descriptions of how to use the code can be found in the comments.

# Reference
